# Advanced Research in Food Digestion

**DOI:** 10.3390/foods10010122

**Published:** 2021-01-08

**Authors:** Ana Andrés, Ana Heredia

**Affiliations:** Institute of Food Engineering for Development, Universitat Politècnica de València, Camino de Vera, s/n 46022 València, Spain; anhegu@tal.upv.es

Food digestion is the key process for delivering nutrients and bioactive compounds to the body. It is well known that dietary factors such as the chemical form of the nutrient, food matrix structure, industrial processing and home preparation can play a significant role in digestibility and bioavailability. Foods are, in general, multiphase mixed dispersed systems in which the organization of nutrients within the food matrix and their physical state give rise to food matrices with specific structures and properties. Besides the food composition, processing also has a key role in promoting ingredients’ interactions (i.e., protein–lipid interactions in the extrusion process). Interactions among nutrients in the same food matrix and among foods in the same meal can also occur and modulate the hydrolysis, bioabsorption and bioavailability of food nutrients and bioactive compounds. On the other hand, the same food can be differently digested depending on gastrointestinal (i.e., reduction in gastrointestinal secretions, alterations in the permeability of the intestinal membrane, etc.) or systemic factors (i.e., age, sex, ethnicity, genotype, physiological state, chronic or infectious diseases, etc.) of the host. Therefore, the functionality associated to the end-digestion metabolites as anti-inflammatory, anti-tumoral or antioxidant agents will depend on both food-inherent properties and gastrointestinal track functionality of the host. 

This Special Issue gathers eight valuable scientific contributions, including one review article and seven original research works, mainly dealing with the mechanisms involved in vegetal food matrices’ digestion, especially of cereals, legumes, seeds and their foodstuffs based on them. The contribution of their end-metabolites to the reduction in metabolic syndrome prevalence-associated diseases such as obesity, high blood pressure, high blood triglycerides, low levels of HDL (high density lipoprotein) cholesterol and insulin resistance is also reported by means of cell culture assays and in vivo and/or in vitro digestion studies. Finally, evidence of nanoencapsulation as a controlled delivery system of carotenoids in the gastrointestinal tract together with the anti-nutrient effect of food stabilizers taking part in food formulations are also discussed in this Special Issue. 

Holland et al. reviewed the composition and organization of polysaccharides taking part in plant tissue and the main mechanisms by which they modulate nutrient release from plant tissues along the human gastrointestinal tract. Some of the most relevant methods currently used to characterize the food matrix and cell walls are also described [1]. Calvo-Lerma et al. pointed out both the role of the structural matrix of chia products (seeds, whole flour, partially defatted flour and sprouts) and intestinal conditions of pH and bile salts’ concentration on the in vitro proteolysis, lipolysis, calcium and polyphenols bioaccessibility [2]. The results evidenced that germination promotes the hydrolysis of chia proteins, making them completely digestible, and causes a significant increase in polyphenols and calcium concentration in chia sprouts. In addition, the particle size and treatments such as defatting or milling had a significant impact on the digestibility of macronutrients and extractability of calcium and polyphenols. Intestinal conditions, however, only play a significant role on the extent of lipolysis of chia. Rojas-Bonzi et al. [3] investigated the in vitro digestion kinetics of breads varying in dietary fiber content and composition and its influence on the glycemic response due to its physicochemical properties in the gastrointestinal tract. Parallelly, they performed an in vivo study with portal vein catheterized pigs to confirm the reliability of the in vitro assay as a predictor of in vivo events. These authors concluded that the in vitro model can provide valuable information about the hydrolysis starch rate. On the other hand, the incorporation of promising new food ingredients based on extruded legume-plus-cereal mixes on the lipid metabolism in rats was investigated by Rubio et al. [4]. Briefly, the inclusion of extruded mixes in obesogenic diets gave, as a result, lowered liver cholesterol, triglycerides and plasma low-density lipoprotein values. In addition, both the inclusion of extruded mixes and the use of obesogenic diets resulted in significantly different long-chain fatty acid profiles in liver and visceral fat compared to the control. Ruiz et al. studied the beneficial health effects of protein fractions from legumes on obesity-associated metabolic disorders [5]. Concretely, they demonstrated the ability of pea vicilin protein hydrolysates resulting from in vitro gastrointestinal digestion to modulate the mRNA expression levels of markers of differentiation and glucose uptake and metabolism in the cell line 3T3-L1 preadipocytes subline. For their part, Zielinski et al. assessed the role of phenolics compounds on the angiotensin-converting enzyme (ACE) inhibitory activity in fermented buckwheat flours and biscuits based on them [6]. The results demonstrated that lactic acid fermentation caused a decrease in ACE inhibitory activity as compared to the non-fermented flour. In addition, the baking process significantly reduced the ACE inhibitory activity of biscuits obtained from fermented flours, whereas digestion significantly increased this property as long as phenolic compounds were released. Therefore, it can be concluded that the cumulative action of those phenolic acids and flavonoids released after digestion is responsible, in part, for the ACE inhibitory activity of buckwheat biscuits.

David et al. reported that carrageenan and xanthan gum interact with food proteins, hindering proteolysis along the gastrointestinal tract [7]. Chocolate milk drinks containing these food anionic stabilizers were digested in vitro, mimicking adults and children’s gastrointestinal conditions and proteomic analyses were performed. LC-MS proteomic analyses revealed that stabilizer addition significantly reduced the bioaccessibility of milk-derived bioactive peptides with differences in liberated peptide sequences arising mainly from their location. Finally, emulsion-based delivery systems have been widely stated as an effective strategy to increase the bioaccessibility of relevant bioactives compounds, and specifically those of low hydrophobicity. In recent years, however, advantages of nano-emulsions over conventional emulsions have been pointed out. Thus, Teixé-Roig et al. aimed to evaluate the effect of pectin addition (0, 1 and 2%) on the physico-chemical stability of oil-in-water nano-emulsions containing β-carotene and its release along in vitro digestion [8]. Their results highlighted the potential of adding pectin to β-carotene nano-emulsions to enhance their functionality by efficiently preventing the compound’s degradation and increasing the in vitro bioaccessibility.

In summary, the Special Issue “Advanced Research in Food Digestion” evidences the great relevance of digestion studies to elucidate the extent of the healthy benefits attributed to foods and the implications of food matrix properties and human digestion physiology conditions on food’s digestive fate. At the same time, the complementarity between in vitro and in vivo approaches to face the current challenges associated to food digestion studies is pointed out by some authors. It is also possible to conclude that omics techniques have brought new opportunities for better understanding relevant food digestion events as pointed out in some contributions. 
